# Supplementary material for: IFN-β is a macrophage-derived effector cytokine facilitating the resolution of bacterial inflammation
Source: Nat Commun. 2019 Aug 2;10:3471. doi: 10.1038/s41467-019-10903-9 (PMC6677895; doi:10.1038/s41467-019-10903-9)
Supplement: Supplementary file 1 — Supplementary Information [file 41467_2019_10903_MOESM1_ESM.pdf]

Supplementary Materials

IFN-β is a macrophage-derived effector cytokine facilitating the resolution of bacterial inflammation

Senthil K. Satyanarayanan[1,#], Driss El Kebir[2,#], Soaad Soboh[1,#], Sergei Butenko[1,~], Meriem Sekheri[2,~], Janan Saadi[1], Neta Peled[1], Simaan Assi[1], Amira Othman[2], Sagie Schif-Zuck[1], Yonatan Feuermann[3], Dalit Barkan[1], Noa Sher[4], János G. Filep[2*], and Amiram Ariel[1*]

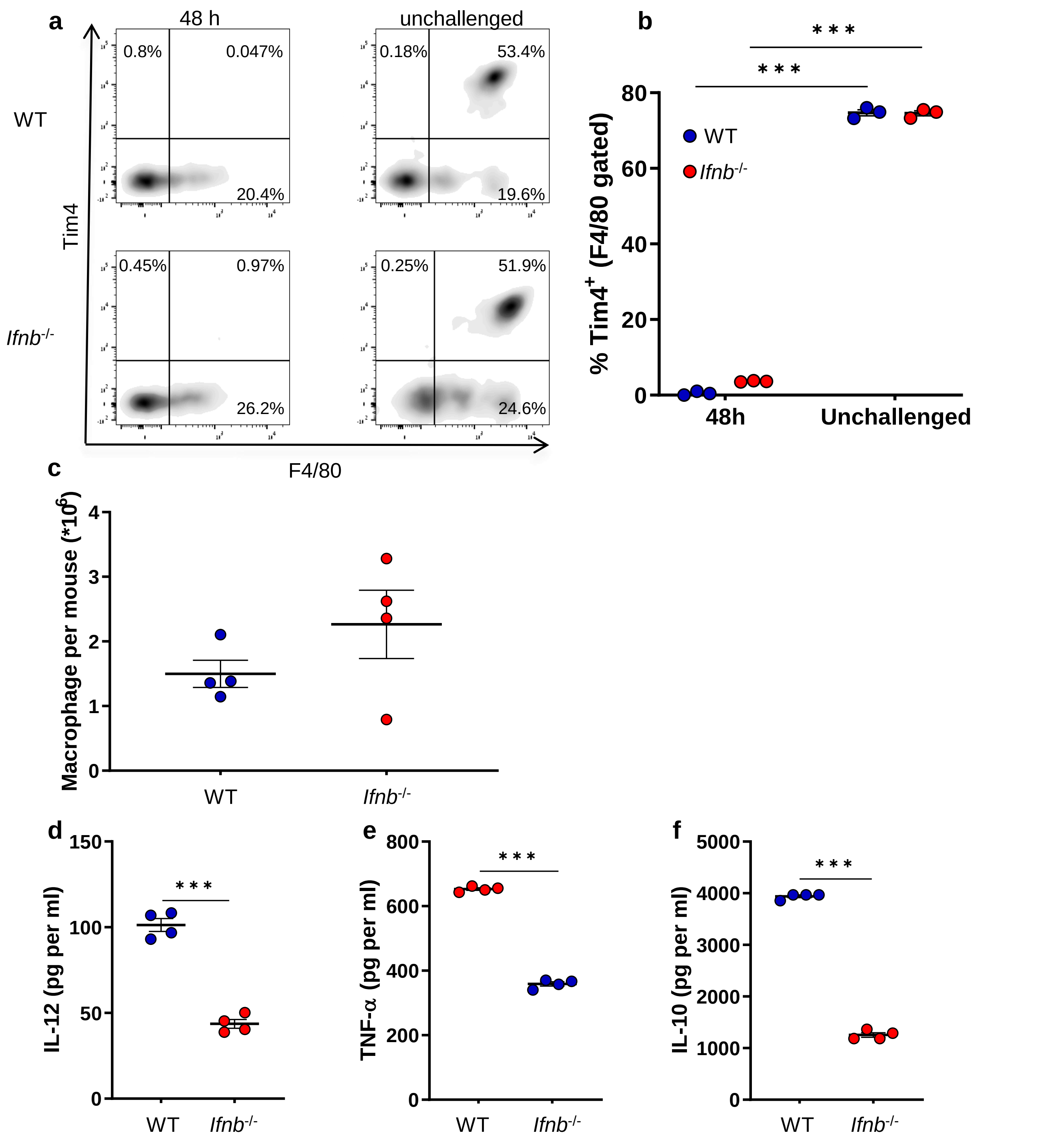

Supplementary Figure 1. Resolution phase macrophages from zymosan A peritonitis are essentially devoid of resident Tim4$^+$ macrophages, and IFN-β deficiency does not affect resident macrophage numbers and origin, but reduces cytokine production. Peritoneal cells were recovered from WT or *Ifnb*$^{-/-}$ mice 48 h PPI or prior to challenge, enumerated and immunostained for F4/80 and Tim4, and the percentage of F4/80$^+$Tim4$^+$ macrophages was analyzed by flow cytometry. (**a**) Representative density plots. (**b**) Values are means ± SEM from 3-5 mice. (**c**) Total resident macrophages numbers. (**d-f**) Macrophage secretion of IL-12 (**d**), TNF-α (**e**), and IL-10 (**f**) after 48 h culture *ex vivo* without challenge. Results are means ± SEM from 4 mice. * P< 0.05, *** P< 0.005 (Student`s t-test). Source data are provided as a Source Data file.

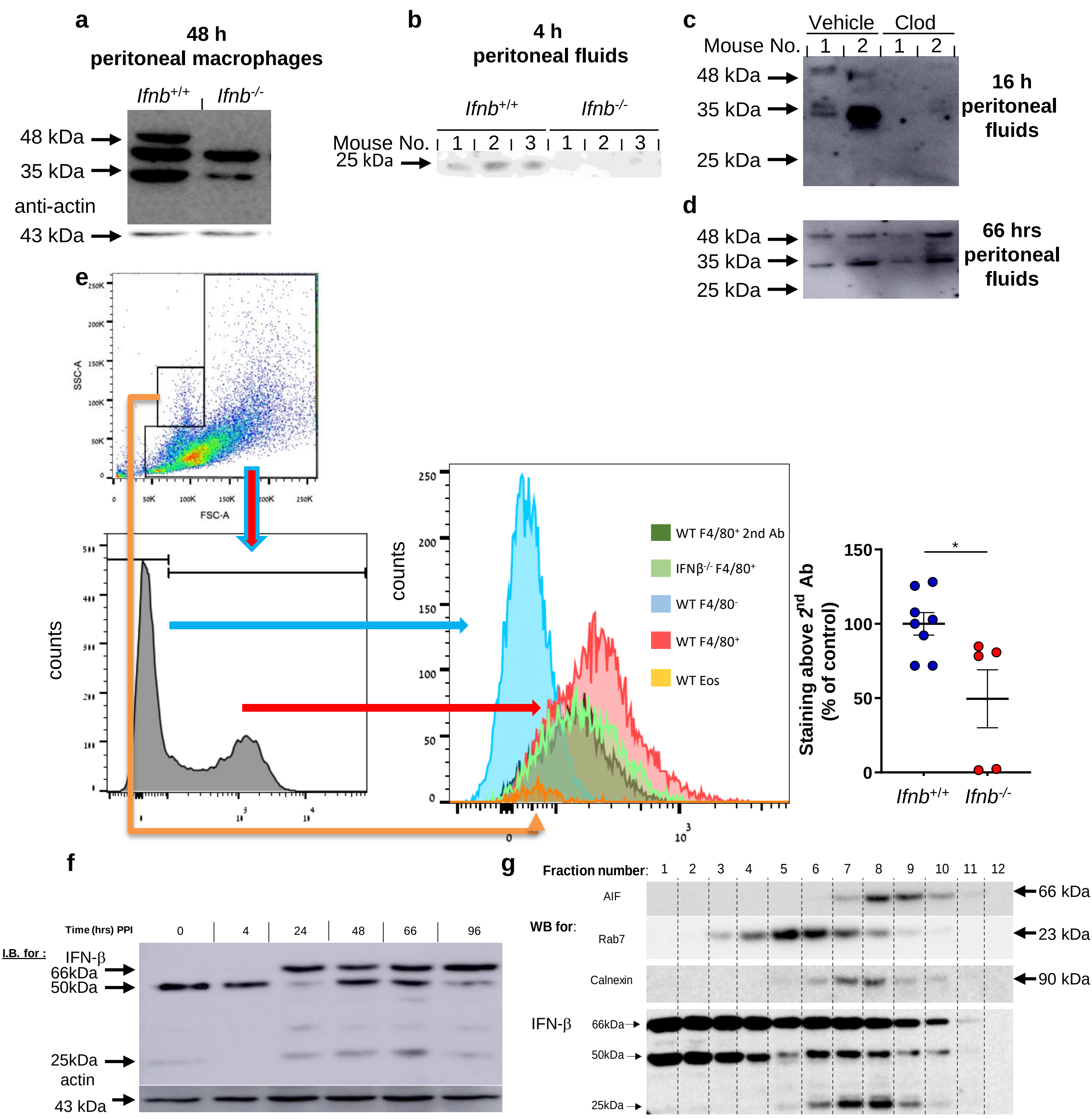
a
48 h
peritoneal macrophages
Ifnb+/+
Ifnb-/-
48 kDa
35 kDa
anti-actin
43 kDa
b
4 h
peritoneal fluids
Ifnb+/+
Ifnb-/-
Mouse No. 1 2 3 1 2 3
25 kDa
c
Vehicle
Clod
Mouse No. 1 2 1 2
48 kDa
35 kDa
25 kDa
16 h
peritoneal fluids
d
48 kDa
35 kDa
25 kDa
66 hrs
peritoneal fluids
e
SSC-A
FSC-A
counts
counts
WT F4/80+ 2nd Ab
IFNβ-/- F4/80+
WT F4/80-
WT F4/80+
WT Eos
Staining above 2nd Ab
(% of control)
Ifnb+/+
Ifnb-/-
f
Time (hrs) PPI
0
4
24
48
66
96
I.B. for :
IFN-β
66kDa
50kDa
25kDa
actin
43 kDa
g
Fraction number: 1 2 3 4 5 6 7 8 9 10 11 12
WB for:
AIF
Rab7
Calnexin
IFN-β
66kDa
50kDa
25kDa
66 kDa
23 kDa
90 kDa

Supplementary Figure 2. IFN-β is expressed as a 50-66 kDa protein in murine macrophages but secreted primarily as a 25-35 kDa protein. Peritoneal exudates were collected from *Ifnb*$^{+/+}$ or *Ifnb*$^{-/-}$ mice 48 (**a**) or 4 h (**b**) PPI and macrophages and peritoneal fluids, were immunoblotted separately for IFN-β. Results are representatives for 4 experiments.

(**c-d**) *Ifnb*$^{+/+}$ mice were injected with clodronate-containing liposomes (Clod) or control vehicle-containing liposomes (Vehicle) 24 h prior to zymosan A administration and peritoneal fluids were recovered at 16 h PPI (**c**). Alternatively, clodronate or vehicle liposomes were injected at 24 h PPI and peritoneal fluids were recovered at 66 h PPI (**d**). Then, peritoneal fluids were run by SDS page and blotted for IFNβ. Blots are representative for 3 independent experiments (N=3 for each treatment).

(**e**) Peritoneal exudates were recovered from *Ifnb*$^{+/+}$ or *Ifnb*$^{-/-}$ mice 48 h PPI. The cells were immunostained for F4/80, fixed, permeabilized, and immunostained for IFN-β. Upper panel shows the gating strategy for eosinophils and other immune cells. The latter were analyzed as F4/80^{+} macrophages (lower panel, right marker) and F4/80^{-} cells (lower panel, left marker). The histograms on the right side show IFN-β levels in the F4/80^{-} (blue line) and eosinophil (orange line) populations from WT mice, as well as 2nd antibody alone (dark green) and the staining of macrophages from *Ifnb*$^{-/-}$ mice (light green). The graph on the right indicates the specific expression of IFN-β in WT mice compared to *Ifnb*$^{-/-}$ mice. Representative plots (left panels) and means ± SEM from 7 *Ifnb*$^{+/+}$ and 5 *ifnβ*$^{-/-}$ mice. * P< 0.05 (Student`s t test).

(**f**) F4/80^{+} macrophages were isolated from peritoneal fluids of unchallenged mice or 4-96 h PPI and lysed. Equal protein amounts were subjected to SDS-PAGE, transferred to PVDF membranes and blotted for IFN-β or actin as control. Blots are representative for 3 independent experiments (N=2-3 mice for each time point).

(**g**) RAW264.7 macrophages were incubated with apoptotic Jurkat cells for 24 h, lysed and organelles were separated on an OptiPrep density gradient with ultracentrifugation. The resulting fractions were subjected to SDS-PAGE and blotted for IFN-β, as well as for AIF, Rab7, or Calnexin that served as indicators for mitochondria, secretory vesicles and ER, respectively. Blots are representative for 2 independent experiments. Source data are provided as a Source Data file.

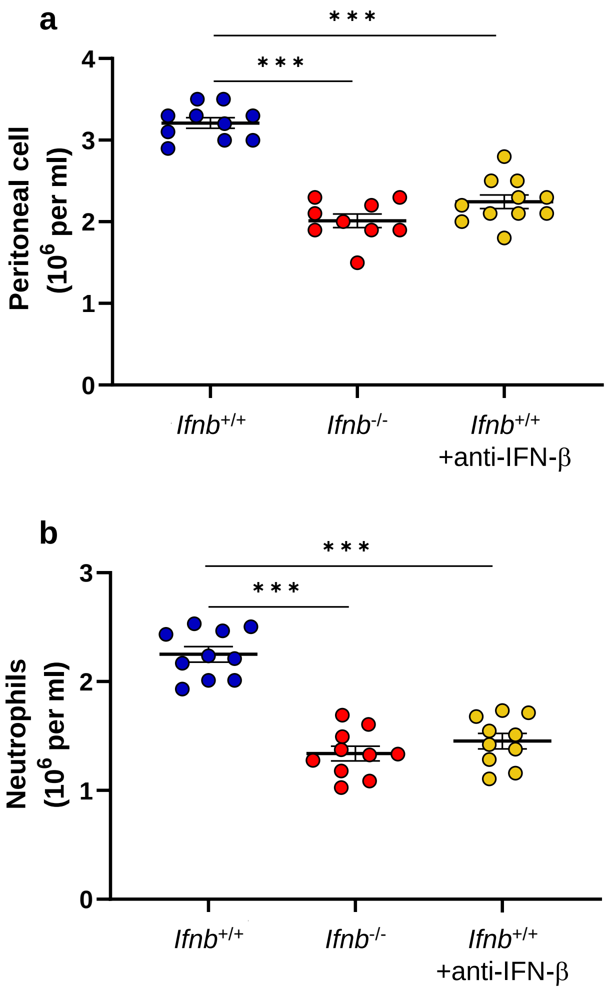

Supplementary Figure 3. IFN-β deficiency limits PMN infiltration to affected tissues during inflammation. *Ifnb*$^{+/+}$ or *Ifnb*$^{-/-}$ mice were injected with zymosan A alone or with anti-IFN-β antibodies (1 µg/mouse). At 4 h, peritoneal exudates were recovered, enumerated and immunostained for Ly-6G, Ly-6C and F4/80. PMN were defined as Ly-6G^{+}F4/80^{-} cells. Total number of cells (**a**) and PMN count (**b**). Values are means ± SEM for 9-11 mice per group. *** P < 0.005 (Tukey`s HSD). Source data are provided as a Source Data file.

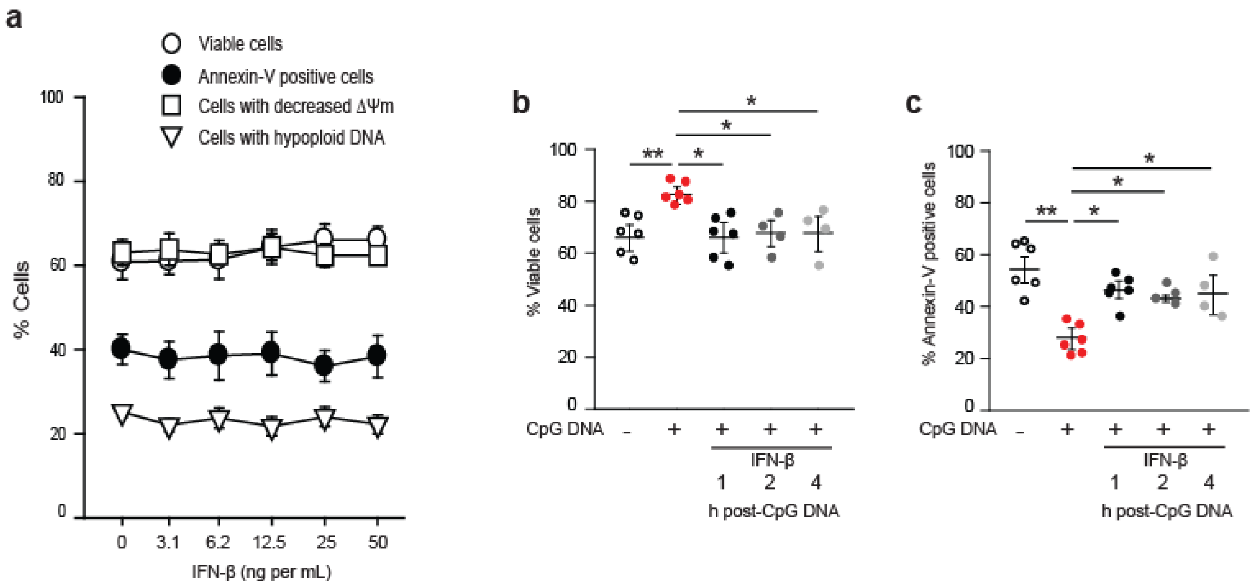

Supplementary Figure 4. IFN-β treatment reverses CpG DNA-induced survival of human neutrophils. (a) Neutrophils (5x10^{6} cells per ml) were cultured with varying concentrations of human recombinant IFN-β or (**b, c**) with CpG DNA (1.6 µg per ml) followed by addition of IFN-β (50 ng per ml) at 1, 2 or 4 h post challenge with CpG DNA. After additional 24 h culture, cell viability, annexin-V staining, nuclear DNA content and mitochondrial transmembrane potential (ΔΨm) were analyzed by flow cytometry. Results are means ± SEM of 4-6 experiments with different blood donors. * P< 0.05, ** P<0.01 (Dunn`s multiple contrast hypothesis test). Source data are provided as a Source Data file.

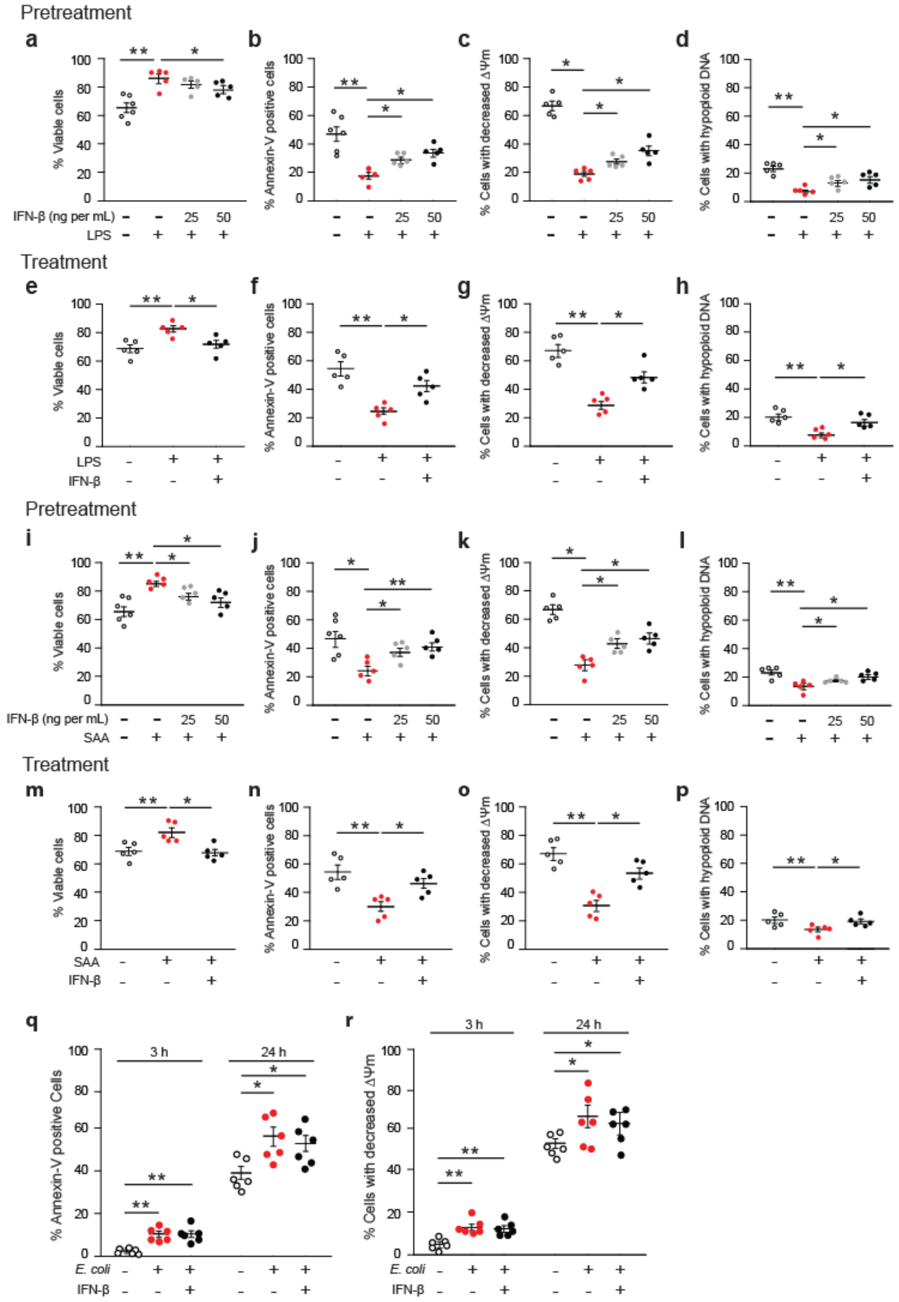

Supplementary Figure 5. IFN-β reverses LPS or SAA-induced survival of human neutrophils, but does not promote phagocytosis-induced neutrophil apoptosis. Neutrophils ($5x10^6$ cells per ml) were cultured with human recombinant IFN-β (50 ng per ml) for 10 min (**a-d, i-l**) then challenged with LPS (1 µg per ml) (**a-d**) or serum amyloid A (SAA, 10 µg per ml) (**i-l**) or were first challenged with LPS (**e-h**) or SAA (**m-p**) for 60 min then treated with IFN-β (50 ng per ml). After additional 24 h culture, cell viability, annexin-V staining, mitochondrial transmembrane potential (ΔΨm), and cell with hypoploid nucliwere analyzed by flow cytometry. (**q-r**) Neutrophils were cultured with human recombinant IFN-β (50 ng per ml) for 10 min, and then *E. coli* was added to the cells at a ratio of 1:7. Development of apoptosis (annexin-V staining and cells with decreased ΔΨm) was assessed at 3 and 24 h post-IFN-β. Results are mean ± SEM of 5-6 experiments with different blood donors. * P< 0.05, ** P<0.01 (Dunn`s multiple contrast hypothesis test). Source data are provided as a Source Data file.

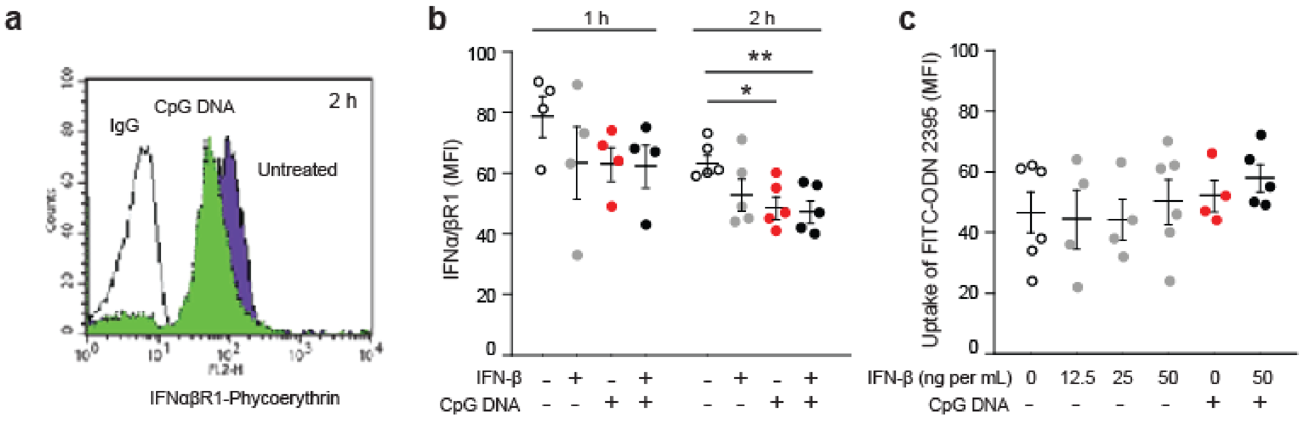

Supplementary Figure 6. CpG DNA downregulates IFNα/βR1 expression on human neutrophils, whereas IFNβ does not hamper ODN uptake. (**a-b**) Neutrophils (5x10^{6} cells per ml) were pretreated with human recombinant IFN-β (50 ng per ml) or vehicle for 10 min and then challenged with CpG DNA (1.6 µg per ml) or vehicle for 1 or 2 h. Surface IFNα/βR1 expression was assessed by flow cytometry (representative plots in **a**) and expressed as MFI after correction based on staining with an isotype-matched irrelevant antibody (calculated averages in **b**). Data are mean ± SEM for 4-5 experiments with different blood donors. *P<0.05, **P<0.01 (Dunn`s multiple contrast hypothesis test).

(**c**) Neutrophils were pretreated with human recombinant IFN-β for 10 min, challenged with CpG DNA (1.6 µg per ml) for 30 min and then uptake of FITC-labeled ODN 2395 was assessed at 30 min by flow cytometry. Data are mean ± SEM for 4-6 experiments with different blood donors. Source data are provided as a Source Data file.

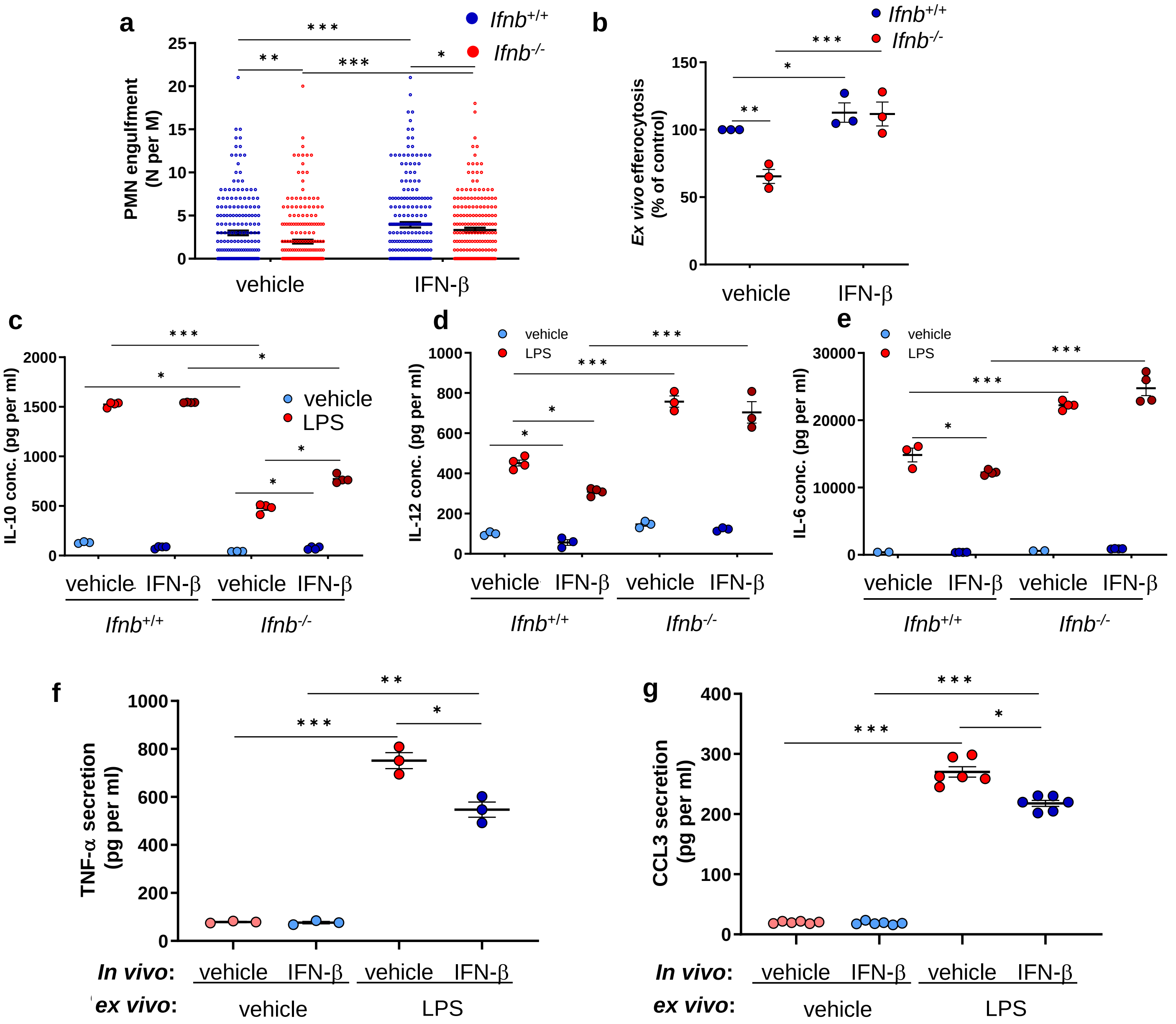

Supplementary Figure 7. IFN-β treatment enhances macrophage reprogramming *in vivo*. (a-b) *Ifnb*$^{+/+}$ or *Ifnb*$^{-/-}$ mice undergoing peritonitis were injected I.P. with IFN-β (20 ng/mouse) or vehicle at 24 h PPI and peritoneal macrophages were recovered at 48 h PPI. In separate experiments, peritoneal macrophages were recovered from *Ifnb*$^{+/+}$ or *Ifnb*$^{-/-}$ mice at 48 h PPI and incubated with CypHer-labeled apoptotic Jurkat cells at a ratio of 1:3. After 4 h, unbound cells were washed. All macrophages were stained with Hoechst 33342 and FITC-phalloidin, the average number of engulfed apoptotic cells in each macrophage were calculated (**a**) and normalized to control (**b**). Results are means ± SEM representative from n=206, 234, 211, and 224 macrophages for vehicle-*Ifnb*$^{+/+}$, vehicle-*Ifnb*$^{-/-}$, IFN-β-*Ifnb*$^{+/+}$ and IFN-β-*Ifnb*$^{-/-}$, respectively (a) and n=523, 475, 335, and 360 macrophages for vehicle- *Ifnb*$^{+/+}$, vehicle- *Ifnb*$^{-/-}$, IFN-β-*Ifnb*$^{+/+}$ and IFN-β-*Ifnb* $^{-/-}$, respectively (b). * P< 0.05, ** P<0.01, *** P< 0.005 (Tukey`s HSD).
(**c-g**) Alternatively, isolated *Ifnb*$^{+/+}$ (**c-g**) or *Ifnb*$^{-/-}$ macrophages (**c-e**) were challenged with LPS (1 μg/ml) or vehicle for 24 h and culture supernatants were analyzed for IL-10 (**c**), IL-12 (**d**), IL-6 (**e**), TNF-α (**f**) or CCL3 (**g**) using standard specific ELISAs. Results are means ± SEM representative from 3 experiments. * P< 0.05, ** P<0.01, *** P< 0.005 (Tukey`s HSD). Source data are provided as a Source Data file.

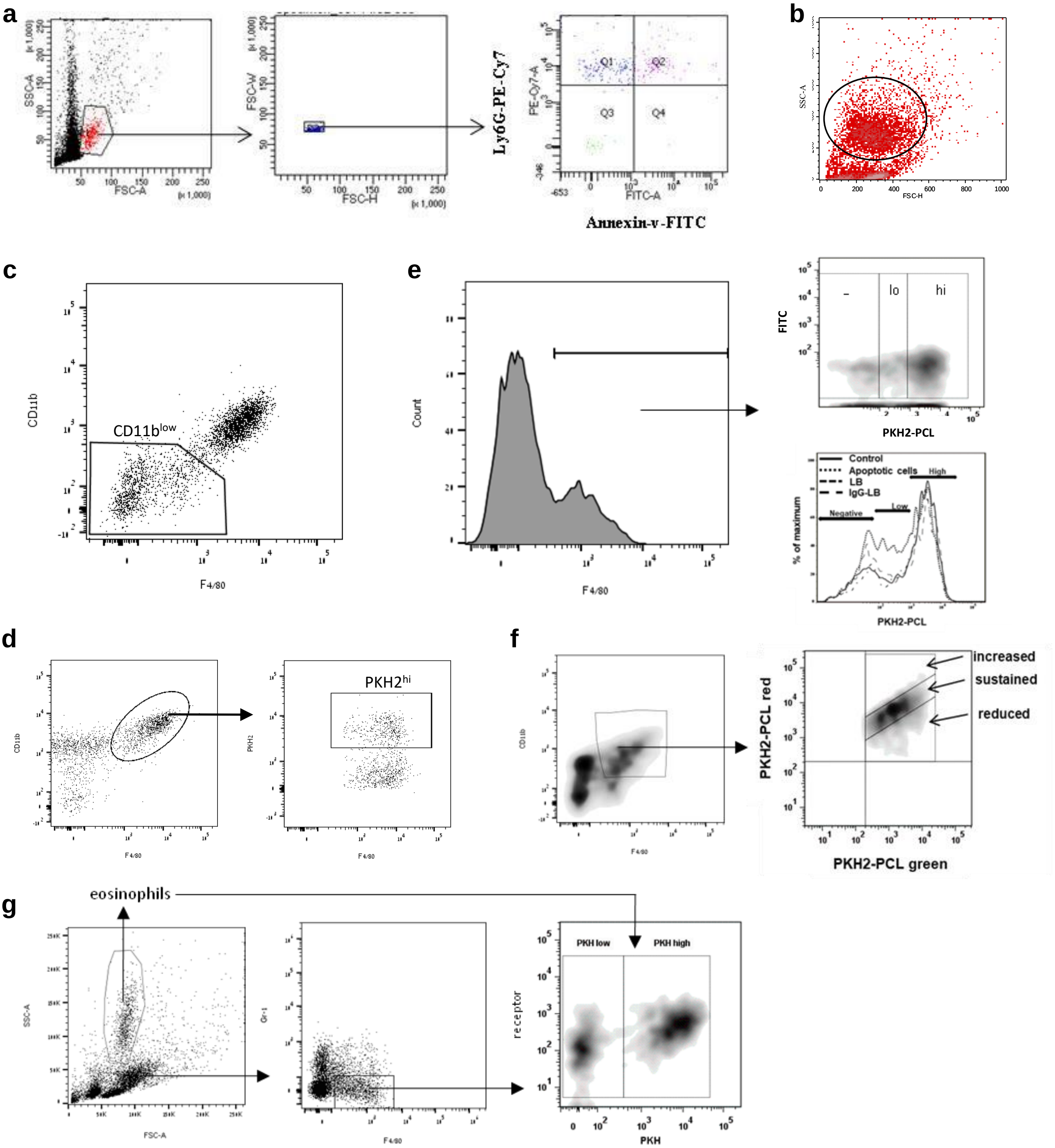

Supplementary Figure 8. Gating strategies used in this manuscript. a. Gating strategy to sort apoptotic neutrophils (Ly6G$^+$/Annexin V$^+$) from bronchoalveolar lavage in mice presented on Figure 2i, Figure 3h and Figure 8h. b. Gating strategy to assess isolated human neutrophils presented on Figure 4d-k, Figure 5c-f, Supplementary Figure 4a-c, Supplementary Figure 5a-r and Supplementary Figure 6a-b. Gating for neutrophils were confirmed by positive staining of the cells with an anti-CD66b antibody. c. Gating strategy for Figure 7g. d. Gating strategy for Figure 9a-b. e. Gating strategy for Figure 9c-g. f. Gating strategy for Figure 9 i-j. g. Gating strategy for Figure 9 k-l.

Supplementary Discussion

Clinical trials have documented the beneficial effects of IFN-β therapy, albeit with highly variable individual patient's response, in multiple sclerosis, a demyelinating and chronic inflammatory disease [1]. By contrast, IFN-β secreted by stromal cells was found to block FAS-induced apoptosis in (unspecified subsets of) T cells, leading to perpetuation of T cell infiltration and chronic inflammation in rheumatoid arthritis [2]. Contradictory case reports suggest that IFN-β therapy either may improve symptoms of preexisting rheumatoid arthritis or may induce polyarthritis in patients with multiple sclerosis who are genetically predisposed to develop arthritis [3]. Since IFN-β-mediated rescue from FAS-triggered apoptosis is essential for the maintenance of memory T cells during the resolution phase, timing of IFN-β therapy may also be critical. It is plausible that the effects of IFN-β therapy will differ in neutrophil-driven inflammatory conditions and autoimmune disorders. This notion is further supported by the findings that IFN-β activates STAT1, but not STAT3 or 5, in T cells to inhibit apoptosis [4], while our results show STAT3, rather than STAT1, is essential for the inhibition of PMN survival.

Supplementary references

1. Panitch, H. et al. Randomized, comparative study of interferon beta-1a treatment regimens in MS: The EVIDENCE Trial. Neurology 59, 1496-1506.(2002)

2. Scheel-Toellner, D. et al. Type I interferons inhibit the resolution of chronic inflammation. Symp Soc Exp Biol 52, 277-288.(2000)

3. Levesque, M.C., Ward, F.E., Jeffery, D.R. & Weinberg, J.B. Interferon-beta1A-induced polyarthritis in a patient with the HLA-DRB1*0404 allele. Arthritis Rheum 42, 569-573.(1999)

4. Pilling, D. et al. Interferon-beta mediates stromal cell rescue of T cells from apoptosis. Eur J Immunol 29, 1041-1050.(1999)
